# Severe falciparum malaria in young children of the Kassena-Nankana district of northern Ghana

**DOI:** 10.1186/1475-2875-6-96

**Published:** 2007-07-27

**Authors:** Abraham R Oduro, Kwadwo A Koram, William Rogers, Frank Atuguba, Patrick Ansah, Thomas Anyorigiya, Akosua Ansah, Francis Anto, Nathan Mensah, Abraham Hodgson, Francis Nkrumah

**Affiliations:** 1 Navrongo Health Research Centre, P.O. Box 114, Navrongo, Ghana; 2 Noguchi Memorial Institute for Medical Research, UG, Box 25, Legon, Accra, Ghana; 3 Naval Medical Research Centre, Malaria Program, Silver Spring, Maryland, USA

## Abstract

**Study design:**

Severe falciparum malaria in children was studied as part of the characterization of the Kassena-Nankana District Ghana for future malaria vaccine trials. Children aged 6–59 months with diagnosis suggestive of acute disease were characterized using the standard WHO definition for severe malaria.

**Results:**

Of the total children screened, 45.2% (868/1921) satisfied the criteria for severe malaria. Estimated incidence of severe malaria was 3.4% (range: 0.4–8.3%) cases per year. The disease incidence was seasonal: 560 cases per year, of which 70.4% occurred during the wet season (June-October). The main manifestations were severe anaemia (36.5%); prolonged or multiple convulsions (21.6%); respiratory distress (24.4%) and cerebral malaria (5.4%). Others were hyperpyrexia (11.1%); hyperparasitaemia (18.5%); hyperlactaemia (33.4%); and hypoglycaemia (3.2%). The frequency of severe anaemia was 39.8% in children of six to 24 months of age and 25.9% in children of 25–60 months of age. More children (8.7%) in the 25–60 months age group had cerebral malaria compared with 4.4% in the 6–24 months age group. The overall case fatality ratio was 3.5%. Cerebral malaria and hyperlactataemia were the significant risk factors associated with death. Severe anaemia, though a major presentation, was not significantly associated with risk of death.

**Conclusion:**

Severe malaria is a frequent and seasonal childhood disease in northern Ghana and maybe an adequate endpoint for future malaria vaccine trials.

## Background

Globally, an estimated 350–500 million clinical malaria episodes occur annually; most of these are caused by infection with *Plasmodium falciparum *with more than one million deaths each year. About 60% of the clinical episodes and more than 80% of the deaths occur in young children in Africa, south of the Sahara, where malaria accounts for 25–35% of all outpatients visits, 20–45% of hospital admissions and 15–35% of hospital deaths [1,2]. Despite the introduction in recent years of more rational antimalarial regimens and the increasing use of the most rapidly parasiticidal artemisinin derivatives [3,4], the malaria risk and mortality has not seen significant reductions yet [5,6]. Studies on factors associated with increased risk of developing severe malaria and death, may provide additional understanding of the course of severe malaria, and, eventually, lead to improved case management, and the development of drugs and vaccines for malaria.

Moreover, this requires the establishment of appropriate case definitions and meaningful trial endpoints for future testing in endemic sites [7]. Several studies, at various sites in Africa, have attempted to delineate the epidemiology of clinical malaria, and the data have shown significant variability across various transmission zones [8-12]. There is, therefore, a need for more site-specific data in order to appreciate the complete clinical and epidemiological picture needed for efficient testing of candidate malaria vaccines and other control tools in different endemic sites. Young children with severe malaria enrolled in order to study the course of severe malaria in the Kassena-Nankana district of northern Ghana, a site being characterized for future malaria vaccine trials.

## Methods

### Study setting

The study was carried out in the Navrongo War Memorial Hospital (NWMH) located in the Kassena-Nankana District (KND) of northern Ghana. The KND lies in the sahelian savannah and covers about 1,674 square kilometres of land with a population of approximately 143,000 under continuous demographic surveillance [13]. The area is characterized by two distinct seasons; a rainy season from June to October and a hot dry season from November to May. Annual rainfall averages 850 mm and means daily temperature ranges from 20°C to 40°C. Malaria transmission is perennial with distinct seasonal patterns. The peak malaria transmission coincides with the period of major rains while the dry season has low rates of malaria infection [14]. Infection is attributed mostly to *P. falciparum *with *Anopheles funestus *and *Anopheles gambiae *as the principal vectors [15]. The estimated annual entomological inoculation rates [15], parasite prevalence [14], malaria attack rates [16] and antimalaria efficacy rates [17] have been well studied.

### Study design and participants

All children between six and 59 months of age, who were admitted at the NWMH with diagnosis suggestive of acute disease, from August to December 2002 and from May 2003 to April 2004, were evaluated for inclusion in the study. Criteria for diagnosis and enrolment included the standard WHO definition for severe malaria [18,19]. Those who satisfied the selection criteria including residence in the KND and whose parents voluntarily gave informed consent were enrolled. Parents were interviewed about the presenting symptoms and study physicians documented findings of clinical examination including weight and vital signs. In addition, the course of illness, including daily vital signs and peripheral blood parasitaemia, were documented for each study child from the time of enrolment to the time of exit from the hospital. Patients were seen daily while on admission and on day 7, 10 and 14 following discharge from the hospital. Those who were alive on day 14 after admission were considered to have survived the acute severe malaria episode. Parents of surviving children were interviewed at home on days 7, 10 and 14 to determine whether their child or ward remained in good health. Participants completed the study by satisfying all study entry requirements and providing all specimens and interviews as provided for by the protocol. This report describes the clinical and laboratory characteristics as well as the prognostic indicators for children admitted with severe malaria.

### Laboratory procedures

At enrolment, peripheral malaria blood smears, full blood count and blood lactate were done for all participants. While on admission, blood smears were taken for daily parasitological examinations until two consecutive smears were aparasitaemic, and on days 7 and 14 from the day of enrolment. Thick and thin blood films were made, thin film fixed with methanol and both thin and thick film stained with 10% Giemsa and examined for malaria parasites. Parasite density was measured as the number of parasites per 200 leucocytes on a thick film and converted into parasites per microlitre of blood based on the participants total white cell count obtained at enrolment. Ten percent of both positive and negative slides were randomly selected and read by an independent microscopist as a quality control check. Two hundred high power fields of the thick films were examined at 1,000× magnification before assigning a negative result. Whole blood specimens were analysed for full blood count using automated ABX Micros 60-OT haematology analyser; serum creatinine, blood glucose and transaminases levels were determined using Micro lab 200, and blood lactate using YSI lactate analyzer.

### Clinical management

Children were treated using a uniform protocol based on standard recommendations[19] consisting of parenteral quinine at 20 mg/kg loading dose followed by 10 mg/kg maintenance dose every 12 hours until the patient was able to swallow, at which point she or he was switched to an oral dose of 10 mg/kg every eight hours to complete a seven day course of treatment. In addition to the specific treatment for *P. falciparum *infection, children received supportive therapy in terms of haemotransfusion for severe anaemia, intravenous glucose for hypoglycaemia and intravenous fluid for severe dehydration. Paracetamol suppositories were administered for pyrexia, diazepam for seizures, antibiotics for bacterial infections, and nasal oxygen for respiratory distress as per standard local practice.

### Approvals

Ethical approval for this study was obtained from the following ethical committees: the Ghana Health Service, the Navrongo Health Research Centre, the Noguchi Memorial Institute for Medical Research and the United States Naval Medical Research Center.

### Data and statistical analysis

All study data were captured on a structured case report form bearing subject demographic and identification numbers. All forms were reviewed before being double entered onto a computer. Statistical analyses were carried out with Epi Info 6.04 (ENSP-Epiconcept-InVS, Corp.) and Stata statistical software (version 7.0, Stata Corporation, College Station, Texas, USA). Continuous and normal distributed data were compared by two-tailed student's t-test and proportions compared with χ^2 ^tests with Yates' correction or Fisher's exact test. Basic statistics were calculated for the baseline characteristics- sex, age group, weight, fever, parasitaemia, presenting symptoms, malaria prevention practices and area of residence. Point estimates using proportions and means, and 95% confidence intervals were computed for the clinical and laboratory features. Significant differences were tested using confidence intervals of the difference or odds ratio and the corresponding (95%) confidence intervals and p-values. A p-value of ≤ 0.05 was considered statistically significant.

## Results

### Baseline characteristics

**Figure 1:**
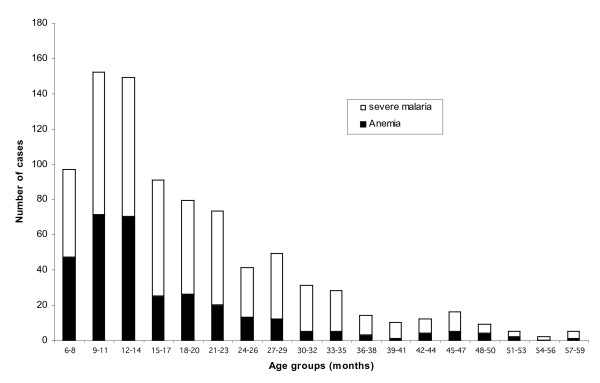
**Age-specific distribution of severe malaria and anaemia in the northern Ghana**. Children aged 6–59 months with severe malaria by WHO criteria (white) and severe anaemia (black).

Out of the total of 1,921 children screened, 73.1% (1405/1921) had a positive parasitaemia and, of these, 61.8% (868/1405) satisfied the criteria for severe *falciparum *malaria. About 56% (490/868) of the total enrolees were males and majority of 76% [661/868] aging below 24 months of age (Figure 1). The average age and weight were respectively 18.9 months (range: 6–59) and 8.9 kilograms [95%CI 8.7, 9.1]. At baseline, the mean haemoglobin and geometric mean parasite density were 6.1 g/dl [range 1.8–13.7] and 26903-parasites/μl [95%CI 22026, 32860] respectively. The proportion with fever (axillary temperature ≥ 37.5°C), thrombocytopaenia and leucocytosis were 77.3% (671/868), 52.1% (452/868) and 47.7% (414/868), respectively. Other enrolment symptoms were vomiting (66.6%), cough (60.7%), shaking chills (57.5%), breathing difficulty (57.4%), lethargy (52.4%), diarrhoea (43.0%), convulsion (39.1%), and inability to eat 38.2%. Reported insecticide-treated bed net (ITN) use and prior malarial chemotherapy were 58.8% (510/868) and 49.5% (430/868) respectively.

## Major clinical and prognostic features

### Cerebral malaria

In all, 47 (5.4%) of the children had cerebral malaria (Blantyre coma score < 3), the frequency of cerebral malaria in the 25–60 months old was 8.7% [95% CI 5.2,13.4] compared to 4.4% [95% CI 2.9, 6.2] in 6–24 months olds. Proportions of boys and girls with cerebral malaria were similar (5% (23/490) vs. 6% (24/378). Fewer children with cerebral malaria had severe anaemia compared to those without cerebral malaria (28%(13/47) vs. 37% (302/816). Again a higher proportion of children with cerebralmalaria had higher respiratory distress than those without (70.2% vs. 21.3%). The mean of haemoglobin, serum lactate and blood glucose among cerebral malaria patients were 7.0 g/dl, 8.5 mmol/l, and 6.2 mmol/l, respectively. The total case fatality ratio among children with cerebral malaria was 29.8% (14/47).

### Severe anaemia

**Figure 2:**
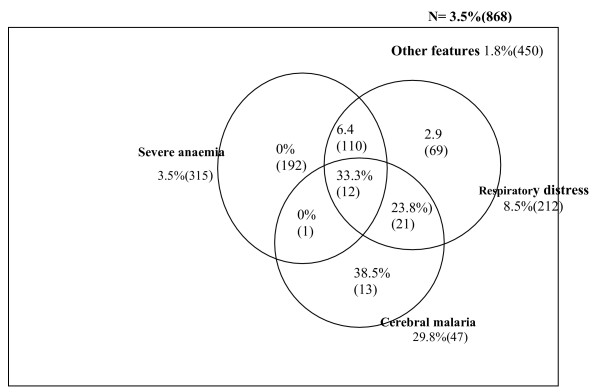
**Venn diagram of proportions, overlap and mortality of severe malaria major clinical subgroups**. Total number of major clinical subgroups in parenthesis and, mortality is given as percentage.

This was the most frequent manifestation, 36.5% (315/863), and was more prevalent in males than females [40.2% vs. 31.6%], in 6–24 month olds than 25–60 olds [39.8 vs. 25.9], Figure 1, and in those not using ITNs than those using ITNs [41.9% vs.32.7%]. The overlap of severe anaemia with cerebral malaria and respiratory distress were 4.1% (13/315) and 38.7% (122/315) respectively (Figure 2). About 87% (274/315) of children with severe anaemia at enrolment received a blood transfusion. The frequencies of severe anaemia in children residing in the irrigated and non-irrigated areas were 29.9(38/127) and 37.6(277/736) respectively. The total case fatality ratio among children with severe anaemia was 3.5%(11/315) but for those who had no other associated severe malaria pathology, total recovery was achieved (Figure 2).

### Respiratory distress

About 24% (212/868) of the participants presented with respiratory distress. Children with respiratory distress were likely to be younger (mean age, 18 months), and had higher geometric mean parasite density (22,026 parasites/microlitre)(Table 1). Again respiratory distress was significantly associated with severe anaemia (OR 3.3 95%CI 2.3, 4.6) and hyperlactataemia (OR 2.3 95%CI 1.5,3.2). The case fatality ratio of respiratory distress was 8.5% [95% 5.1,13.1]. The ratio was significantly increased to 23.8% and 33.3% if the respiratory distress overlapped with cerebral malaria or cerebral malaria plus severe anaemia respectively (Figure 2).

**Table 1:** Characteristics of the major clinical subgroups.

**Parameters**	**Cerebral Malaria (n = 47)**	**Severe Anaemia (n = 315)**	**Respiratory Distress (n = 212)**
**Males, n (%)**	23(48.9)	202(61.8)	117(55.2)
**6–24 months, n (%) mean age**	21.6 (13.1)	16.5(10.0)	17.9(11.3)
**Mean haemoglobin, g/dl (SD)**	7.0(2.6)	3.8(0.8)	5.2(2.3)
**Mean blood lactate, mmol/L(SD)**	8.5(5.7)	5.8(4.1)	6.6(4.9)
*GMPD, parasites/μL	15223	15361	22026
**Mean blood glucose, mmol/L (SD)**	6.2(3.6)	5.0(1.8)	5.1(2.1)
**Hypoglycaemia, n (%)**	6(12.8)	8(2.5)	18(8.5)
**Hyperlactataemia, n (%)**	34(72.3)	142(45.1)	101(47.6)
**Hyperparasitaemia, n(%)**	8(17.0)	34(10.8)	33(15.6)
**Hyper pyrexia, n (%)**	1(2.1)	12(3.8)	28(13.2)

* GMPD = geometric mean parasite density

Other severe malaria manifestations at enrolment were multiple or prolonged convulsions 21.6% (187/864), hyperlactataemia 33.4% (249/746), hyperparasitaemia 18.5% (161/868) and hypoglycaemia 3.2% (24/761).

### Factors associated with malaria death

The overall study case fatality ratio was 3.5 (30/868). The ratio was similar in males and females (3.3% vs. 3.7%) and in 6–24 and 25–59 month's olds (3.3 vs. 3.9%). Their baseline presentations were: cerebral malaria 46.7% (14/30), severe anaemia 36.7% (11/30), respiratory distress 60%(18/30) and hyperlactataemia 61.5(16/26). Bivariate analysis (Table 2) showed that cerebral malaria, hypoglycaemia, respiratory distress and hyperlactaemia were the risk factors associated with most deaths, but logistic regression analysis showed that only cerebral malaria OR= 12.9(95% CI 4.1,40.8) and hyperlactataemia OR = 1.2 (95%CI 1.1, 1.3) were risk factors associated with falciparum malaria death.

**Table 2:** Prognostics indicators at the time of enrolment

Outcome	Prevalence,n(%)	Fatalities, n(%)	OR	χ^2^	95% CI	P-value
Cerebral malaria	47 (5.4)	14(29.8)	21.3	103.3	8.7,50.7	0.000
Severe anaemia	315 (36.5)	11(3.5)	1.02	0.02	0.45,2.28	0.88
Respiratory distress	212 (24.4)	18 (8.5)	4.9	20.7	2.1,11.3	0.000
Hyperlactataemia	290 (33.4)	19 (6.6)	3.6	20.7	1.6,8.6	0.000
Hypoglycaemia	33 (3.8)	4(12.1)	4.3	7.7	1.0,13.5	0.005

### Incidence of severe malaria

To estimate severe malaria incidence in the population, a mid year population of children 6–59 months old in the district from the Navrongo demographic surveillance database and the total cases from May 2003 to April 2004 were used. In all, there were 16,298 children (aged 6 to 59 months) and 560 severe malaria cases giving an overall incidence of 0.034 cases per year. The incidence was highest in the age group, 6–11 months and lowest in the 48–59 month old (Table 3).

**Table 3:** Age-specific incidence of severe malaria in the KND (2003–2004)

**Age group (Months)**	**Mid year population (% of total)**	**Number of cases (% of total)**	**Estimated incidence of severe malaria.**
6–11	1632 (10.0)	136 (24.2)	0.083
12–23	3474 (21.3)	262 (46.8)	0.075
24–35	3536 (21.7)	105 (18.8)	0.030
36–47	3816 (23.4)	41 (07.3)	0.011
48–59	3840 (23.6)	16 (02.9)	0.004
Total	16298 (100)	560 (100)	0.034

### Seasonal variation

**Figure 3:**
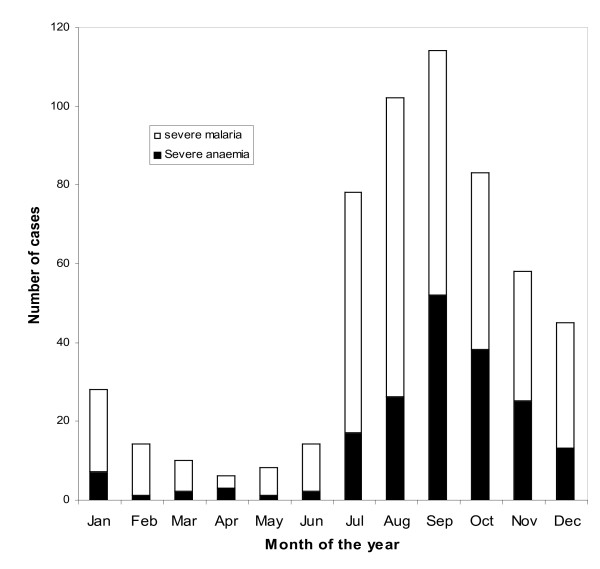
**Seasonal prevalence of severe malaria and anaemia in the Kassena-Nankana district of Ghana**. WHO criteria of severe malaria (white) and severe anaemia (black) by month of the year.

Of the 560 cases of severe malaria enrolled during the one year period [May 2003 to April 2004], 70.4% (394/560) were enrolled in June-October (the high transmission season) and 29.4%(166/560) in November-May (low transmission season). The highest number of cases, 127 (22.7%) were enrolled in September and the lowest, 6 (1.1%) in April (Figure 3). Though more cases of severe anaemia (72%) occurred during the high transmission season, the proportions of severe anaemia in the high and low seasons were similar (35.5% vs. 33.1% p-value > 0.05). About 71% (22/31) of cerebral malaria cases occurred in the wet season compared to 29% (9/31) in the dry season. The geometric mean parasite density in the high season was 37,876 parasites/μl [95%CI 31,069/46,174] compared to 21,473-parasites/μl [95%CI 15,564/29,624] in the low season. The prevalence of hyperparasitaemia was also greater in the high transmission season than in the low season (22.1% versus 7.4%, p-value < 0.001). The case fatality ratios during the high and low seasons were 4.2% (7/166) and 1.8% (7/394), respectively.

## Discussion

As part of malaria characterization studies in northern Ghana in preparation for malaria vaccine trials severe malaria among young children was studied. The results showed that significant population and numbers of severe *P. falciparum *malaria exist in the Kassena-Nankana District of northern Ghana that may represent an adequate vaccine trial endpoint. Severe malaria, as a vaccine trial endpoint offers several advantages over other endpoints that could be used in future malaria vaccine efficacy trials [20,21]. Even though mortality impact is the most important public health measure of any vaccine efficacy, it represents a smaller proportion of all disease burdens and will, thus, require large sample size in future trials [22]. Also, in many malaria endemic countries, assignment of causes of deaths may present sensitivity and specificity problems that may lead to misclassification and reduction of the statistical power of any trial with mortality as an endpoint[23]. On the other hand, it is difficult to define and differentiate mild malaria and infection as endpoints from many of the other common febrile illnesses in most endemic areas [24,25]. Again the comparative ease of using the established case definitions of severe malaria [18] and the fact that most children diagnosed as having the disease have worse prognoses than those with mild disease makes severe malaria a better trial endpoint. Well-defined severe malaria, therefore, bridges the gap of being common enough for a reduction to be measurable and as important as possible to predict malaria-associated mortality in endemic sites [21]. For instance, in the recent study in Mozambique, RTSS/AS02A vaccine showed a significant protection against severe malaria [26].

The current study showed that over a period of fifteen months, 868 severe cases were recruited, with most of the cases (76%) being children between 6–24 months old, with a significant decreasing trend towards increasing age (Figure 1). As in other settings the predominant manifestation was severe malarial anaemia. Other clinical and laboratory manifestations were also consistent with what have earlier been reported in other endemic settings [9-12]. Using the total number of cases of severe malaria in one year and the mid year population of children at risk in the study area, the annual incidence of severe malaria was estimated to be 3.4% (95% CI 3.1,3.7): ranging from 0.4% in 48–59 months old to 8.3% in 6–12 months olds (Table 3). This is significantly higher than the 2.3% reported in a similar setting in another part of West Africa [21], even though this represent only hospital cases instead of census as in that study [21]. This suggesting that this finding may even be an underestimation, as some children might have died at home during the period since almost all cases of untreated severe and complicated malaria are fatal.

The overall case fatality ratio in this study was 3.5%; this was similar by gender (3.3% vs. 3.7%) and by the age groups (3.3% vs. 3.9%). The independent prognostic indicators were cerebral malaria and hyperlactataemia confirming the fact that in severe malaria, neurological involvement and metabolic dysfunction are the factors that are most associated with poor outcome [9,27,28]. Severe anaemia, though the most frequent presenting feature, was a poor predictor of death [29,30], perhaps because most severe malaria anaemia children received timely blood transfusion. Despite this, severe anaemia will be an important vaccine trial endpoint because of its frequency, especially in children and because of the ease with which it can be measured with certainty in field situations.

Further significant seasonal variation of severe malaria was recorded over the period. The highest prevalence occurred in the months of July to December (during the rainy season). The observed pattern points to the fact that increase in vector breeding following the raining season is responsible for the upsurge in the malarial cases and supports an earlier transmission studies [15]. In malaria endemic areas, majority of malaria death and morbidity occur during the peak transmission seasons. As such, the intermittent preventive treatment approaches to malaria control during this intense period [31] could impact on reducing disease burden. Such preventive measures could reduce the risk of severe malaria and subsequently minimize its effect as a vaccine trial endpoint. For instance the results showed that ITNs usage was high; patients not using ITNs had higher incidence of severe anaemia and cerebral malaria compared to those using ITNs. These considerations need to be factored in when estimating sample size for future vaccine studies.

## Conclusion

Major conclusions can be drawn from this study. First, severe malaria in the area occurs frequently in the first 30 months of life, the predominant feature being severe anaemia. Most of the severe malaria occurs in the high transmission and short period, which suggest that the area can effectively support intervention studies including vaccine trials in the prevailing short, but intensive, transmission season.

## Authors' contributions

AO, KK, WR, FN: conception and design, and data acquisition, analysis and interpretation. Manuscript drafting and revision for the intellectual content.

FA, FA, AT: conception and design, and acquisition and interpretation of data.

PA, AA, NM, AH: acquisition and interpretation of data, and manuscript preparation.
